# Indications for upper gastrointestinal endoscopy before bariatric surgery: a multicenter study

**DOI:** 10.1007/s00464-022-09656-2

**Published:** 2022-10-06

**Authors:** Hussein Abdallah, Mehdi El Skalli, Hussein Mcheimeche, Biagio Casagranda, Nicolò de Manzini, Silvia Palmisano

**Affiliations:** 1 Department of Medical, Surgical and Health Sciences, University of Trieste, Strada di Fiume, 447, 34149 Trieste, Italy; 2 Centre Hospitalier Universitaire Montpellier, Université de Montpellier 1, Montpellier, France; 3 Department of Surgery, Al Zahraa Hospital University Medical Center, Beirut, Lebanon; 4 Surgical Clinic Division, Cattinara Hospital, ASUGI, 34149 Trieste, Italy

**Keywords:** Preoperative endoscopy, Bariatric surgery, Helicobacter pylori infection, Upper gastrointestinal diseases

## Abstract

**Background:**

The role of preoperative upper gastrointestinal endoscopy before bariatric surgery is still debated, and a consensus among the international scientific community is lacking. The aims of this study, conducted in three different geographic areas, were to analyze data regarding the pathological endoscopic findings and report their impact on the decision-making process and surgical management, in terms of delay in surgical operation, modification of the intended bariatric procedure, or contraindication to surgery.

**Methods:**

This is a multicenter cross-sectional study using data obtained from three prospective databases. The preoperative endoscopic reports, patient demographics, Body Mass Index, type of surgery, and Helicobacter pylori status were collected. Endoscopic findings were categorized into four groups: (1) normal endoscopy, (2) abnormal findings not requiring a change in the surgical approach, (3) clinically important lesions that required a change in surgical management or further investigations or therapy prior to surgery, and (4) findings that contraindicated surgery.

**Results:**

Between 2006 and 2020, data on 643 patients were analyzed. In all of the enrolled bariatric institutions, preoperative endoscopy was performed routinely. A total of 76.2% patients had normal and/or abnormal findings that did not required a change in surgical management; in 23.8% cases a change or a delay in surgical approach occurred. Helicobacter pylori infection was detected in 15.2% patients. No patient had an endoscopic finding contraindicating surgery.

**Conclusions:**

The role of preoperative UGE is to identify a wide range of pathological findings in patients with obesity that could influence the therapeutic approach, including the choice of the proper bariatric procedure. Considering the anatomical modifications, the incidence of asymptomatic pathologies, and the risk of malignancy, we support the decision of performing preoperative endoscopy for all patients eligible for bariatric operation.

**Graphical abstract:**

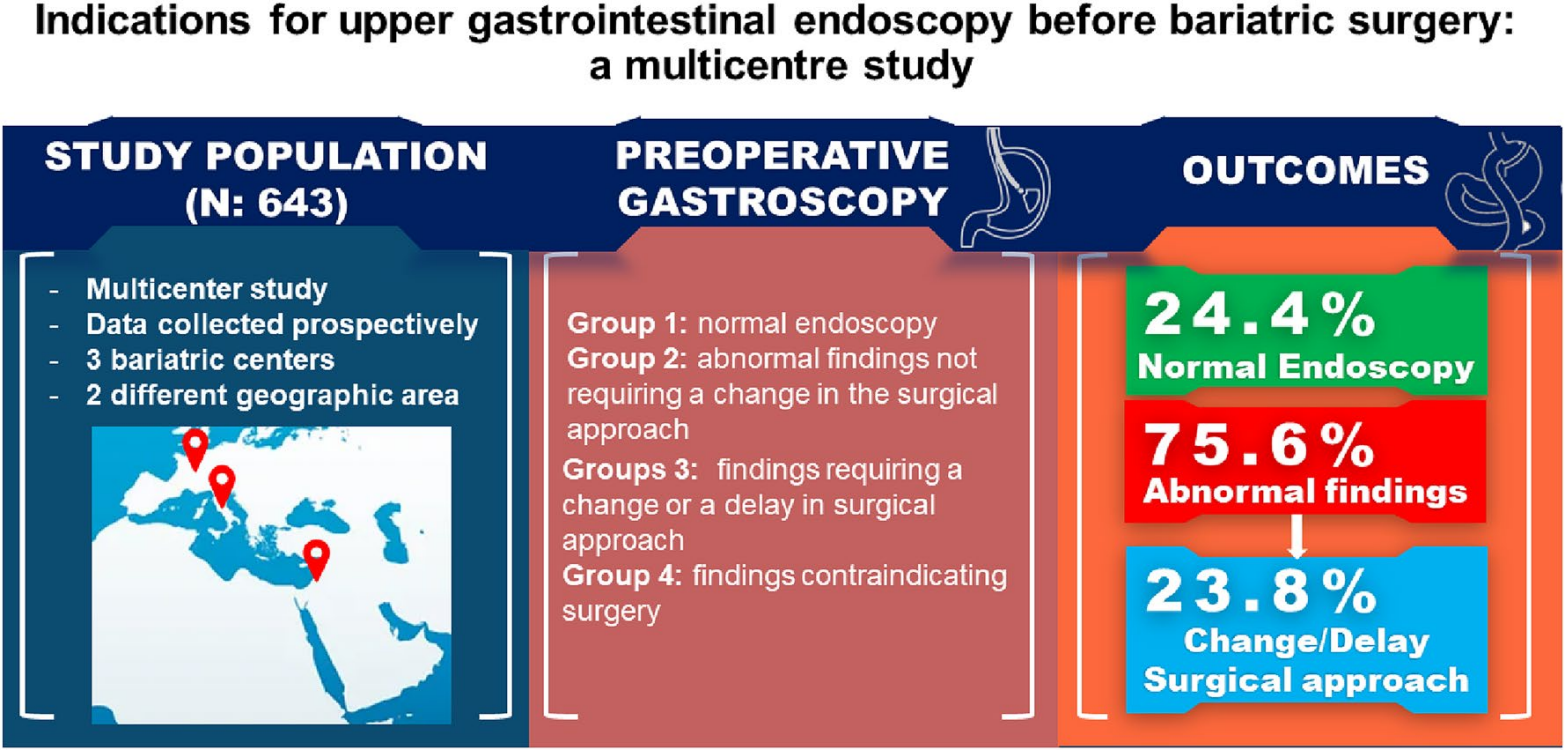

Obesity is a major public health issue, affecting an increasing number of countries worldwide because of its prevalence, costs, and health effects. In the United States of America from 1999 to 2018 the prevalence of obesity increased from 30.5 to 42.4%, and the prevalence of severe obesity increased from 4.7 to 9.2% [1]; in Europe, in 2014, its average prevalence reached 15.9% with associated increased morbidity and mortality [2]. As compared with conventional therapy, bariatric surgery has proven to be effective for the treatment of clinically severe obesity, reducing the overall mortality [3, 4] with improvement or resolution of associated comorbidities and quality of life [5–9].

Before the bariatric procedure, the patient with obesity proposed for surgery should undergo multiple clinical-instrumental assessments and counseling in order to establish the suitability for the intervention. Since obesity is considered an important risk factor for the development of gastrointestinal (GI) disorders, and malignancies and bariatric surgery will change both anatomy and physiology of the gastrointestinal tract [9], upper gastrointestinal endoscopy (UGE) was considered a significant tool in the preoperative work-up. In addition, pathological endoscopic findings could affect the surgical decision-making process and modify the planned surgical procedure.

In the scientific community, there is still no full agreement on the role of the preoperative endoscopy in mainly asymptomatic patients. The Italian Society for Bariatric Surgery and Metabolic Disorders (S.I.C.OB) [10] and the American Society for Metabolic & Bariatric Surgery (ASMBS) [11] suggest an individualized approach based on the presence of symptoms. On the other hand, German guidelines [12] recommend UGE for all patients who are candidates for bariatric surgery. The IFSO 2020 position statement [13] mentions the possibility to perform upper endoscopy in all patients undergoing bariatric surgery (symptomatic or asymptomatic), as abnormal findings were found in a pooled mean 15.4% of asymptomatic patients. Analyzing data reported by the IFSO 2020 position statement and ASMBS Standards of Practice Committee [11], this consideration is particularly relevant in regions where the background incidence of significant gastric and esophageal pathologies is high, such as in Asian populations.

The aims of our study, conducted in three different geographic areas, were to (1) obtain additional and significant data on the role of the preoperative UGE; (2) analyze the incidence of pathological endoscopic findings; and (3) report the change in surgical approach in terms of delay in surgical management, modification of the intended bariatric procedure or contraindication to bariatric surgery.

## Materials and methods

A multicenter cross-sectional study *performed using* data obtained from three prospective databases was carried out. All patients with obesity willing for bariatric surgery were briefed on the purpose of the study, and the informed consent was obtained from all individual participants enrolled. As it was a retrospective study on data collected prospectively, no further approval was required from the Institutional Review Committee or the local ethics committee.

All preoperative UGE reports prior to primary bariatric surgery between June 2006 and September 2020 in three bariatric institutions in different geographical areas were recorded. The collected data included patient demographics, Body Mass Index (BMI), UGE findings, and Helicobacter pylori (H. pylori) status.

Multiple gastric biopsies were routinely taken from the antrum and corpus to rule out H. pylori infection or further abnormal findings. All patients with H. pylori infection were offered a standard therapy and, in all cases, a stool antigen test was performed. GERD was defined by linking typical patient’s symptoms and endoscopic finding, according with Los Angeles classification.

The endoscopic findings were classified into four groups according to the classification proposed by Sharaf et al. [14]. Group 1 included patients with normal UGE; group 2 was composed of patients who had abnormal findings that did not require a change in the surgical approach; groups 3 and 4 included clinically important lesions: group 3 consisted of patients who had findings that required a change in surgical management or further investigations or therapy prior to surgery (e.g., H. pylori infection, mucosal/ submucosal mass lesions, ulcers, severe erosive esophagitis, gastritis, and/or duodenitis, Barrett’s esophagus (BE), bezoar, hiatal hernia, peptic stricture, Zenker’s or esophageal diverticula, arteriovenous malformations), while group 4 included endoscopic findings that contraindicated surgery (e.g., upper gastrointestinal cancers and varices).

We also asked all participating centers which were their position on the role of preoperative UGE (whether performed routinely or in presence of symptoms only). The incidence of endoscopic pathological findings and changes in surgical decision-making process were recorded.

## Results

Among 669 patients who underwent primary bariatric surgery, 26 patients with missing endoscopic or pathological reports were excluded from the study; thus, the total number of patients enrolled in our analysis was 643. The median age was 43,5 years (range 13–75 years) and the median BMI was 44 kg/m^2^ (range 30–70 kg/m^2^). There were 208 (n = 208/643, 32.3%) males and 435 (n = 435/643, 67.7%) females. In all of the enrolled bariatric institutions, UGE was performed routinely. The most frequently performed bariatric procedure was RYGB (n = 314/643, 48,8%;); LSG, One Anastomosis Gastric Gypass (OAGB), and Gastric Banding (LGB) were performed in 282 patients (n = 282/643, 43.9%), in 44 patients (n = 44/643, 6.8%) and in 3 patients (n = 3/643, 0.5%), respectively. RYGB was the most frequent procedure performed in the Italian center, as according to its specialists it provides better results in term of weight loss and co-morbidity resolution/improvement in the short and long term. All procedures were performed laparoscopically.

No complications were reported in any UGE procedure. Abnormal endoscopic findings were present in 75.5% of patients (n = 486/643).

H. pylori infection was histologically detected in 98 patients (n = 98/643, 15.2%) and only 3 patients had concurrent peptic ulcer. In all cases, an eradication treatment was prescribed and its efficacy was evaluated against the negativity of the fecal antigen test.

Patients with large hiatal hernia (n = 17/643, 2.6%) were explored intraoperatively and all had a concurrent hiatoplasty: 12 cases (n = 12/17, 70.6%) underwent a direct repair, while in 5 patients (n = 5/17, 29.4%) a posterior partial fundoplication was performed. Esophagitis was found in 78 patients (12.1%) and more than half were categorized in grade A according to the Los Angeles classification system, in 62 cases GERD was associated, and in 2 patients BE was detected. One benign tumor and 18 polyps were discovered, and in one case, a well-differentiated gastric neuroendocrine tumor (NET) was detected. There were 11 patients (1.7%) with metaplastic (chronic) atrophic gastritis. All other endoscopic findings are provided in Table 1.

**Table 1:** Endoscopic findings. Several patients presented more than one abnormal finding

UGE findings	Number/Percent (*n* = 643)
Abnormal UGE	486/75.5%
*Gastritis (Sydney classification)* Mild Moderate Severe	317/49.3% 277/87.4% 29/9.2% 11/3.4%
Hiatus hernia Hiatal insufficiency or small hiatal hernia ≤ 3 cm Large hiatal hernia > 3 cm with/or without paraesophageal hernia	168/26.1% 151/89.9% 17/10.1%
H. Pylori infection	98/15.2%
*Esophagitis (Los Angeles classification)* Grade A Grade B Grade C Grade D	78/12.1% 58/74.4% 11/14.1% 6/7.7% 3/3.8%
GERD	62/9.6%
Erosions	44/6.8%
*Polyps* Gastric Duodenal Esophageal	18/2.8% 15/83.3% 1/5.6% 2/11.1%
Duodenitis	11/1.7%
Peptic Ulcer	11/1.7%
Metaplastic (chronic) atrophic gastritis	11/1.7%
*Tumors* Benign (submucosal gastric lipoma; esophageal squamous papilloma) Malignant (Neuroendocrine tumor—NET G3)	2/0.3% 1/50% 1/50%
Barrett’s esophagus (BE)	2/0.3%
Bezoar	1/0.2%
Glycogenic Acanthosis (esophagus)	1/0.2%

Considering all the pathological findings detected by the preoperative UGE, according with the Sharaf et al. classification [15], on 490 patients (76.2%), the planned surgical procedure was confirmed: 157 (24.4%) cases reported regular gastroscopy (Group 1), and in 333 (51.8%) patients abnormal findings did not require a change in planned surgical management (Group 2). Of the 486 (75.5%) abnormal findings, in 153 (23.8%) cases the surgical plan was changed or delayed (Group 3). No endoscopic findings contraindicating bariatric surgery were reported (Group 4) (Table 2).

**Table 2:** Endoscopic reports and related surgical management

Groups	UGE findings	Number of patients, *N* (%)	Descriptions/Surgical management
Group 1	Normal gastroscopy	157 (24,4%)	Normal and Abnormal findings not requiring a change in surgical management
Group 2	-Mildesophagitis, gastritis and/or duodenitis -Benign polyps -Esophageal webs	333 (51,8%)	
Group 3	-H. pylori -Mass lesions (mucosal/submucosal), Gastrointestinal Stromal Tumors (GIST), Neuroendocrine tumor (NET) -Ulcers (any location) -Severe erosive esophagitis, gastritis and/or duodenitis -Barrett’s esophagus -Bezoar -Hiatal hernia -Peptic stricture -Esophageal diverticula -Arteriovenous malformations	153 (23,8%)	Abnormal findings requiring a change or a delay in surgical approach
Group 4	-Upper GI cancer -Varices	0 (0%)	Contraindication to bariatric surgery

## Discussion

There is still some controversy regarding the indication of performing UGE during preoperative work-up in patients seeking bariatric surgery.

Areas of debate include indications of UGE in non-symptomatic patients and the impact of the endoscopic findings on the surgical procedure plan and outcomes. Having regard to the risk–benefit balance and the low percentage of abnormal findings requiring a change in operative management reported in a recent systematic review [16], the routine use of UGE as a screening tool before any bariatric operation would appear economically and clinically unjustified. These considerations led some surgical societies to suggest an individual-based approach in relation to the presence of symptoms [15, 17] and/or a scheduled bariatric procedure leading to partial gastric exclusion, such as RYGB or OAGB [11]. In our experience, the rate of abnormal endoscopic findings was 75.6% and the most commonly endoscopic abnormality was mild gastritis, which does not require any change or delay in the surgical approach.

However, there are additional considerations to bear in mind. In literature, several studies report a poor correlation between patient’s symptoms and endoscopic abnormalities [14, 18, 19], Not all esophageal, gastric, and duodenal diseases are symptomatic, especially early malignancies. An up to 11.3 times, higher incidence of distal esophageal adenocarcinoma and gastric cardia adenocarcinoma in patients with obesity compared to lean subject was reported [20]. In our experience, eleven patients had metaplastic (chronic) atrophic gastritis, and one patient had NET discovered at preoperative UGE biopsy. These pathological entities are asymptomatic, and the metaplastic (chronic) atrophic gastritis is associated with an increased risk for gastric cancer [21, 22]. In these cases, based on the endoscopic report, instead of the intended surgery (RYGB), LSG was performed. It should also be considered that obesity is a well-known risk factor that has been found to increase the risk of erosive esophagitis, BE, and gastric adenocarcinoma by contributing to the development of GERD [23, 24]. In their study, Ghaderi et al. reported an overall rate of endoscopic abnormalities in asymptomatic patients of 80.2%, and a rate 4.6% of BE with no significant differences between symptomatic and asymptomatic patients [25]. In all these cases, without the preoperative histological report, the surgeon could have chosen inappropriate bariatric interventions, putting the patients at risk of dangerous neoplastic evolution. As reported so far, the UGE findings could change the operative strategy in term of modification the planned intervention.

In addition, several histological findings could be also responsible for surgical delay. In the literature, the most common causes of surgery delay were H. pylori infection, peptic ulcer, and BE. In our cohort, these patients were treated for at least 4–6 weeks with standard therapy. Some patients required repeated endoscopy before operation, and in 5 cases, a modification of the planned bariatric operation was required. In our study, the rates of H. pylori infection and peptic ulcer were 15.3% and 1.7%, respectively. The colonization rates of H. pylori in patients with obesity differ considerably across studies, from 23 to 70% [13, 20, 25–27], and it is considered as a risk factor for active chronic gastritis, gastric ulcer, and gastric malignancy. In the bariatric population, it has also been correlated with postoperative complications, acting as a risk factor for developing marginal ulcer after RYGB and OAGB [28], and with a longer hospitalization and higher readmission rate after LSG [29].

The incidence of BE in the literature varies between 0.2 and 3,1% and reached 7,89% in GERD population [30, 31]; GERD incidence ranges from 10 to 20% in the general population. In our study, the rate of BE was 0.3%, and that of GERD was 9.6%. These variations might be related to population’s characteristics, dietary habits, and the endoscopist’s experience and judgment. Considering the higher incidence of GERD in patients with obesity, the risk of BE is not negligible. As BE with dysplasia is regarded as a precursor to distal esophageal cancer, it was considered a major contraindication for LSG by the 95% experts who attended the last international Conference [32]. On these bases, the presence of BE in the endoscopic report should demand a change in the surgical approach, in terms of delay or modification of the intended operation (from LSG to RYGB) [33, 34].

The aim of the study was to provide a scientific contribution to the field of the preoperative endoscopic assessment based on the experience of three bariatric centers belonging to different geographic areas (Middle East and Europe). This study presents the following limitations: (1) the distribution of patients was not homogeneous among the four types of intervention; (2) the selection bias: RYGB constituted the majority of study patients at baseline, which could have underestimated the percentage of patients in whom the operation type was changed based on the EGD finding; and (3) the retrospective nature of the study.

In conclusion, the role of preoperative UGE is to identify a wide range of pathological findings in patients with obesity that could influence the therapeutic approach, including the choice of the proper bariatric procedure. It is our belief that beyond the one-by-one incidence of the reported endoscopic abnormalities, a more complex reasoning should be established. Firstly, it must be considered that the anatomical changes occurring after bariatric operations, not only specifically after LSG [35–38], could possibly place “healthy” patients with obesity at a higher risk to develop upper gastrointestinal tumors. Secondly, in our experience, the overall incidence of the pathological endoscopic findings associated with a higher risk of gastric and esophageal cancer was 28.7%. Such a rate of undetected endoscopic abnormalities, in case of UGE not performed, is in our opinion not negligible nor acceptable. Thirdly, our data showed that in almost 24% of cases, endoscopic abnormalities led to change or delay the surgical management. This is a substantial information that should not be overlooked as these pre-existing conditions would also affect the postoperative outcomes. Finally, a comprehensive assessment of any organ, in this case the stomach, prior to surgery should be considered a good clinical practice, especially if the planned operation involves an anatomic modification that makes the gastric remnant no longer accessible. Thus, for all these reasons, from our perspective, we support the decision to run UGE for all patients eligible for bariatric operation regardless of symptoms and type of surgery planned.
